# Sabbath Recreations

**Published:** 1859-10

**Authors:** 


### SABBATH RECREATIONS.

Bible believers understand, without any argument, that the Sabbath was intended to be observed as a day of rest, being so called, "because, that in it He (the Almighty) had rested from all his work."

Recreation is not rest, but rest is recreation; a "making over again," literally; it renews, gives fresh life and vigour, and readiness for work again.

Any man knows, who has tried it, that going to the country early in the morning, and roaming about through the woods, in fishing or hunting, or any other form of amusement, during the day, and then returning to the city by horse, or foot, or boat, or carriage, or rail, leaves the body tired, weary, worn out, almost exhausted, quite as much, if not more so, than if the ordinary avocations had been followed.

Let a man take his station at any depot or ferry, on landing at the close of any holiday, and inspect the countenances of those returning, and there will be presented, in most cases, an expression of sadness and weariness, which is amost pitiful, to say nothing of the riotous and drunken, the outlaws, and the brawlers. To such, the Sabbath has been no day of rest, of renewal; it finds them quite as weary as the previous Saturday evening found them, and they wake up on Monday morning as unrested, as unrefreshed, as unrenewed, as on any other day of the week, and wearily enough do they go to work, instead of having that eager alacrity for another week's toil, which a whole day of in-door quiet, bodily and mental, would have secured for them.

The notion, therefore, that an excursion to the country on Sundays, with the excitement of its novelties, has any wholesome effect on mind, or body, or heart, anything invigorating, renewing, life-giving, is a physical and physiological absurdity, the proof of it being any man's own observation and experience. There is no rest in locomotion. There is no rest in mental excitement, and both locomotion and mental excitement are inseparable from those Sunday recreations for the laboring poor, for which some of the penny press, and other less creditable papers, are contending.

The actual practical effect of these Sunday excursions to the country are:

1st. To induce the poor laborer to squander his money for the benefit of grog-sellers and Sabbath-breakers, instead of spending it for the substantial comfort of his wife and children; for if he takes them along, the savings of the whole week are consumed.

2. To rob him of the only day which he can call his own, and of the enjoyment he might derive in quiet communion with his family.

3. To deprive him of the opportunity for the recuperation which is absolutely essential for the healthful, vigorous, and faithful discharge of the duties and labors of the new week.

